# ACDC: a general approach for detecting phenotype or exposure associated co-expression

**DOI:** 10.3389/fmed.2023.1118824

**Published:** 2023-05-19

**Authors:** Katelyn Queen, My-Nhi Nguyen, Frank D. Gilliland, Sung Chun, Benjamin A. Raby, Joshua Millstein

**Affiliations:** ^1^Department of Population and Public Health Sciences, Keck School of Medicine, University of Southern California, Los Angeles, CA, United States; ^2^Division of Pulmonary Medicine, Boston Children's Hospital and Harvard Medical School, Boston, MA, United States; ^3^Channing Division of Network Medicine, Department of Medicine, Brigham and Women's Hospital and Harvard Medical School, Boston, MA, United States; ^4^Division of Pulmonary and Critical Care Medicine, Department of Medicine, Brigham and Women's Hospital and Harvard Medical School, Boston, MA, United States

**Keywords:** gene expression, differential co-expression, asthma, asthma control, inflammation

## Abstract

**Background:**

Existing module-based differential co-expression methods identify differences in gene-gene relationships across phenotype or exposure structures by testing for consistent changes in transcription abundance. Current methods only allow for assessment of co-expression variation across a singular, binary or categorical exposure or phenotype, limiting the information that can be obtained from these analyses.

**Methods:**

Here, we propose a novel approach for detection of differential co-expression that simultaneously accommodates multiple phenotypes or exposures with binary, ordinal, or continuous data types.

**Results:**

We report an application to two cohorts of asthmatic patients with varying levels of asthma control to identify associations between gene co-expression and asthma control test scores. Results suggest that both expression levels and covariances of ADORA3, ALOX15, and IDO1 are associated with asthma control.

**Conclusion:**

ACDC is a flexible extension to existing methodology that can detect differential co-expression across varying external variables.

## 1. Introduction

Differential expression analysis has long been used to test for differences in transcriptional dependencies across conditions, and may explain phenotypic variation in a population. However, differential expression methods study each gene independent of any other and therefore may not capture transcriptional differences due to changes in gene-gene relationships. Differential co-expression methods test for differences in gene covariances, and thus, such approaches may illuminate regulatory mechanisms not identified by differential expression analysis alone (1).

Module-based differential co-expression methods incorporate information about gene connectivity, and assume that the genes within a module are correlated in the general population. These approaches can have good statistical power due to a reduction in “noise” (2), or unrelated variation of individual genes by collapsing related genes into a single feature. Generally, these module-based methods can be distinguished from one another by, (i) whether modules are defined by the user or the method, (ii) if differential co-expression is detected within or between modules, and (iii) how many conditions are assessed. Methods may also detect differential co-expression for gene pairs across the phenotype of interest and then apply *post-hoc* clustering methods to identify co-expressed modules. One highly-cited method, CoXpress, determines differentially co-expressed modules given microarray data (3). By cutting the trees determined by average-linkage hierarchical clustering at a user-defined threshold, genes are split into modules. Then, pairwise correlation coefficients are used to created a distribution of co-expression for each module under two conditions. If these distributions are statistically significantly different from random in one condition and not the other, the module is considered differentially co-expressed.

While many methods exists for binary conditions and a few for greater than two, we are unaware of any module-based differential co-expression approaches designed to detect differences across continuous conditions or multiple types of conditions simultaneously. Here we describe a novel method, **a**ssociation of **c**ovariance for detecting **d**ifferential **c**o-expression (ACDC), to detect differential co-expression across multiple binary, ordinal, or continuous phenotypes or exposures. We report an application to gene expression measured in two independent cohorts of asthmatics to determine whether genes in inflammatory pathways are co-expressed across levels of asthma control.

## 2. Materials and methods

### 2.1. ACDC description

ACDC is designed to detect dependencies between gene-gene co-expression (or connectivity) and a set of external features that can be either exposures or responses. That is, ACDC is applied to test for evidence of association between measures of co-expression and measures of external features. Notably, the external features are not constrained to be categorical, the typical requirement (2), but could be continuous or ordinal.

The concept of covariance can be used to quantify the dependence between two random variables and thus to quantify gene-gene co-expression. It is possible for the covariance of a pair of genes to depend on external features. For example, suppose in a biological pathway, two genes tend to be co-regulated and thus co-expressed, resulting in positive covariance. A perturbation to the pathway could alter that relationship, resulting in a change in co-expression and thus a change in covariance. If candidate perturbagens and the expression of genes in the pathway are measured, ACDC may be applied to detect these types of effects simultaneously for the multiple genes and perturbagens. Using a similar rationale, ACDC could be applied to detect downstream results of pathway perturbations if the affected phenotypes are measured.

Suppose all individuals have measurements for all *M* gene expression features in the set, referred to here as a “module”, and all *P* external features. Assume the vector of *P* external features are distributed as multivariate normal,


(1) $$\begin{array}{l} x={\left(x_{1},x_{2},...,x_{P}\right)}^{T}\sim N\left(\mu_{x},\Sigma_{x}\right) \end{array}$$

with *x*_*p*_ representing each external feature. Though we describe ***x*** as multivariate normal here, we can relax this assumption in practice and allow other distributions and variable types, as in a design matrix.

Suppose the *M* gene expression features are also distributed as multivariate normal with the covariance matrix depending on *x*,


(2) $$\begin{array}{l} g={\left(g_{1},g_{2},...,g_{M}\right)}^{T}\sim N\left(\mu_{g},\Sigma_{g}|x\right) \end{array}$$

where *g*_*j*_ denotes the expression of gene *j*. The covariance matrix can be represented by,



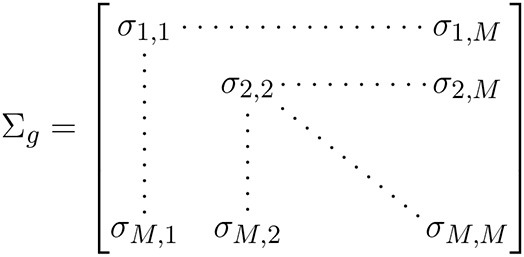



The off-diagonal elements of Σ_*g*_ can be considered measures of co-expression, and (for given values of x) estimated in the conventional way,


(4) $$\begin{array}{l} \begin{array}{c} {\hat{\sigma }}_{j,k}=\frac{1}{N-1}\Sigma_{s}\left(g_{s,j}-\bar{g_{j}}\right)\left(g_{s,k}-\bar{g_{k}}\right). \end{array} \end{array}$$

Note that this is essentially an average over individuals. Letting *s* denote an individual, each contribution is,


(5) $$\begin{array}{l} {\hat{\sigma }}_{j,k}=\left(g_{s,j}-\bar{g_{j}}\right)\left(g_{s,k}-\bar{g_{k}}\right) \end{array}$$

These individual components have approximately the same expectation as the scaled sum, therefore they can also be described as estimators for σ_*j, k*_. We leverage this property to test for dependencies between the covariances and the external features.

We can denote the co-expression profile for a given module as,


(6) $$C=\left(\sigma_{1,2},...,\sigma_{j,k},...\sigma_{(M-1),M}\right);|C|=\left(\begin{array}{c} M\\ 2 \end{array}\right)=G.$$

We are interested in dependencies that may exist between the external features, *x*, and the gene-pair covariances, the off diagonals of Σ_*g*_. If we have a single external feature or a single pair of genes, conventional general linear modeling (GLM) approaches could be used to relate *x* to *C*. For multiple gene pairs and external features, CCA can be applied, or sparse CCA for high dimensional settings. CCA finds min [*G, P*] linear combinations, *a*∈ℝ^*P*^, *b*∈ℝ^*G*^, of *C* and *x*, respectively, that maximize the correlation,


(7) $$\begin{array}{l} \left(a_{1}^{\prime },b_{1}^{\prime }\right)=\text{argmax corr}\left(a_{1}^{T}x,b_{1}^{T}C\right)\text{;}\rho_{1}=\text{corr}\left(a_{1}^{T}x,b_{1}^{T}C\right), \end{array}$$

for example, for the first pair of canonical variables. Note that CCA can be applied even if *G* and/or *P* is equal to one (4). Wilks–Lambda can be used to conduct a joint hypothesis test of whether the correlation coefficients found by CCA are significantly different from zero,


(8) $$\begin{array}{l} \begin{array}{c} H_{0}:\rho_{i}=0,\text{for all}1\leq i\leq \min\;\left[G,P\right]\\ H_{A}:\rho_{i}\neq 0,\text{for some}1\leq i\leq \min\;\left[G,P\right]. \end{array} \end{array}$$

A rejected test implies dependent co-expression, i.e., that there are linear combinations of gene-gene covariances associated with linear combinations of external features.

False discovery rates (FDR) can be computed using the Benjamini–Hochberg (BH) (5) method when multiple modules are tested and parametric assumptions apply. If severe departures from the assumed distributions may be present, permutation-based approaches such as the Millstein and Volfson (MV) FDR (6) method can be used.

and is also available from the CRAN repository, https://cran.r-project.org/web/packages/modACDC/index.html

### 2.2. Datasets

#### 2.2.1. Asthma BRIDGE

The Asthma Biorepository for Integrative Genomic Exploration (ABRIDGE) aimed to bring together data from over 2,700 participants in ongoing (at the time) asthma studies (7). Patients were recruited from six cohorts of the EVE Consortium, a group of 11 academic sites who did genome-wide association studies of asthma (8), and extensive phenotype and genomics data are publicly available.

**Figure 1:**
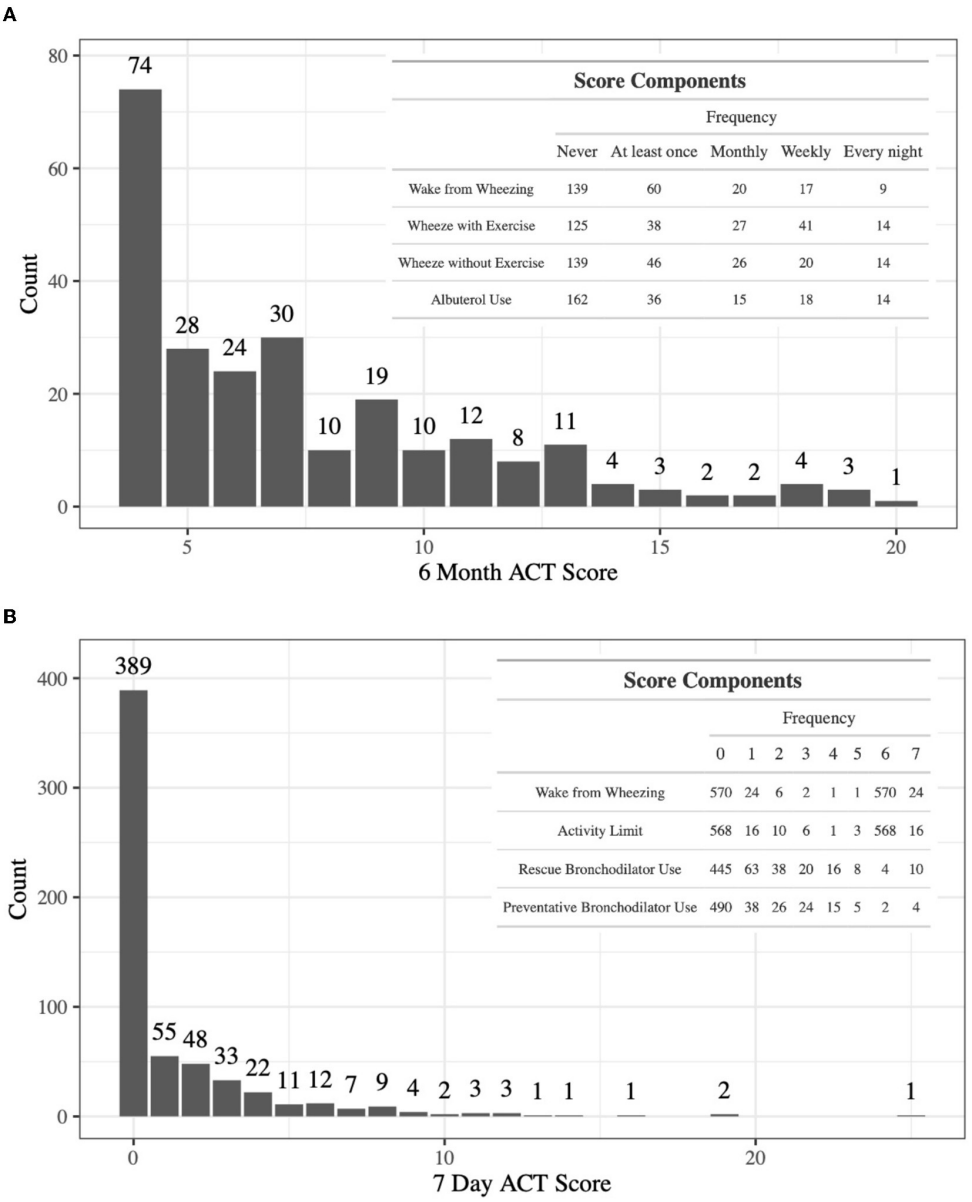
**(A)** The distribution of 6-month ACT scores in ABRIDGE Whole Blood gene expression, with scores being calculated with information about wheezing with and without exercise, patient waking due to wheezing, and the need for rescue medications in the last 6 months. **(B)** The distribution of 7-day ACT scores in CAMP Whole Blood gene expression, with scores being calculated with information about the need for rescue and preventative medications, activity limits, and patient waking due to wheezing in the past 7 days.

The discovery dataset includes gene expression in whole blood from 245 patients with doctor-diagnosed asthma from ABRIDGE (Table 1), profiled using the Illumina HumanHT-12 v4 Expression array. Six-month asthma control test (ACT) scores were calculated from questionnaire responses about wheezing with and without exercise, patient waking due to wheezing, and the need for Albuterol in the last 6 months (range: [4,20]), where higher scores indicate suboptimal control (Figure 1A).

**Table 1:** Patient demographics for ABRIDGE and CAMP cohorts.

	**ABRIDGE (*n* = 245)**	**CAMP (*n* = 604)**
**Age** (years), mean ± sd	22.02 ± 5.22	20.91 ± 2.22
**Age at asthma diagnosis** (years), mean ± sd	4.96 ± 3.87	3.03 ± 2.38
**Sex**, male (%)	121 (49.39)	376 (62.25)
**Race**		
European	34	413
Hispanic/Latino	177	59
Black/African American	0	90
American Indian or Alaska Native	1	4
East/Southeast Asian	0	5
Uncertain or other	33	33
**Data collection site**		
CAMP	0	604
Children's Health Study	107	0
Mexico City Childhood Asthma Study	138	0

The gene expression profile data were normalized via a log2-transformation and quantile-normalization. Duplicate probes were condensed using the largest median absolute deviation, leaving only probes with unique targets. The analysis includes 623 probes with targets annotated for inflammatory response in Gene Ontology.

#### 2.2.2. CAMP

The Childhood Asthma Management Program (CAMP) was a randomized, placebo-controlled clinical trial started in the early 1990s for children with mild to moderate asthma. One thousand and forty-one children were enrolled between 1993 and 1995 at eight clinical centers, and extensive baseline data was collected and is publicly available (GEO accession number GSE22324) (9).

Results from the initial analysis were followed up in an independent dataset that included whole blood gene expression from 604 asthmatics, primarily young adults who were enrolled in CAMP as children (Table 1), profiled using the HumanRef8 v2 BeadChip array. Seven-day ACT scores were calculated using baseline questionnaire responses about rescue and preventative bronchodilator use, activity limits, and frequency of waking due to wheezing in the past 7 days (range: [0,28]), where higher scores indicate suboptimal control (Figure 1B). The same data processing normalization steps were taken as in Asthma BRIDGE.

### 2.3. ABRIDGE and CAMP data analysis

To identify modules of correlated genes, we applied the Partition data reduction method (10, 11), an agglomerative approach that requires the user to specify an acceptable proportion of information loss when collapsing all features to a single measure such as the mean. Selection of the information loss threshold was guided by the aim to maximize information explained in the ACT score while minimizing noise. Further explanation is provided in the Supplementary material (12). We used an information loss constraint of 0.35 which corresponds to a minimum of 65% information from the non-reduced data captured by each new feature, as assessed by the intraclass correlation coefficient (ICC). This reduction threshold resulted in roughly 50% reduction in features when compared to the full dataset (Supplementary Figure 1).

In analyses of individual genes within modules of interest, gene-ACT score relationships were modeled using ordinal logistic regression, adjusting for patient age, race, sex, data collection site, and the first three principal components (PCs), which may capture global dependencies due to cell-type composition and technical artifacts, of each gene expression data set. Associations were identified at the 0.05 FDR level.

To clarify the novel attributes of ACDC, a comparative analysis was conducted in the ABRIDGE cohort using CoXpress. The ACT score was dichotomized at the median value to indicate better vs. worse asthma control to conform to the coXpress requirement of a binary phenotype. The Pearson correlation coefficient was used as the similarity measure, and for module identification the dendrogram was cut at a height of 0.35 for consistency with the Partition approach.

## 3. Results

### 3.1. ACDC in ABRIDGE

ACDC was performed on 65 modules identified by Partition in the ABRIDGE dataset. The results for the top five modules based on BH FDR can be found in Table 2. Evidence suggestive of differential co-expression as determined by CCA Wilks–Lambda *p* ≤ 0.05 was found for two modules including genes NOD-like Receptor Family Pyrin Domain Containing 12 (*NLRP12*), Meteorin Like, Glial Cell Differentiation Regulator (*METRNL*), and Ghrelin And Obestatin Prepropeptide (*GHRL*) in module A (BH FDR = 0.0737), and Adenosine A3 Receptor (*ADORA3*), Arachidonate 15-Lipoxygenase (*ALOX15*), and Indoleamine 2,3-Dioxygenase 1 (*IDO1*) in module B (BH FDR = 0.1569). We also computed the non-parametric, permutation based FDR estimate Millstein–Volfson (MV) to account for departures from the normality assumption by the ACT variable, which is ordinal. However, the results of the MV FDR test are in approximate agreement with the BH FDR results, yielding two modules with evidence of differential co-expression [(Supplementary Figure 2), FDR = 0.0554, 95% CI: (0.0054, 0.5742)].

**Table 2:** Results of CCA analysis between gene-gene covariances and ACT score components for ABRIDGE and CAMP cohorts.

		**ABRIDGE**			**CAMP**	
**Module**	**Genes**	**CCA correlation coefficients**	**CCA** ***p*****-value**	**BH FDR** ***q*****-value**	**CCA correlation coefficients**	**CCA** ***p*****-value**
A	*NLRP12, METRNL, GHRL*	0.3021, 0.1957, 0.0276	0.0012	0.0737	0.1061, 0.0624, 0.0147	0.6823
B	*ALOX15, IDO1, ADORA3*	0.2863, 0.1451, 0.1063	0.0040	0.1569	0.1574, 0.0823, 0.0761	0.0315
C	*IL5RA, PMP22*	0.1860	0.0753	0.9999	0.1595	0.0038
D	*IL17RB, IL6*	0.1740	0.1157	0.9999	0.1303	0.0361
E	*IL16, NLRC3, SLAMF1*	0.2310, 0.1387, 0.0142	0.1195	0.9999	0.0556, 0.0534, 0.0106	0.9893

The top five modules in ABRIDGE out of the 65 are shown for brevity, and the analysis was repeated in the CAMP cohort for these five modules.

**Figure 2:**
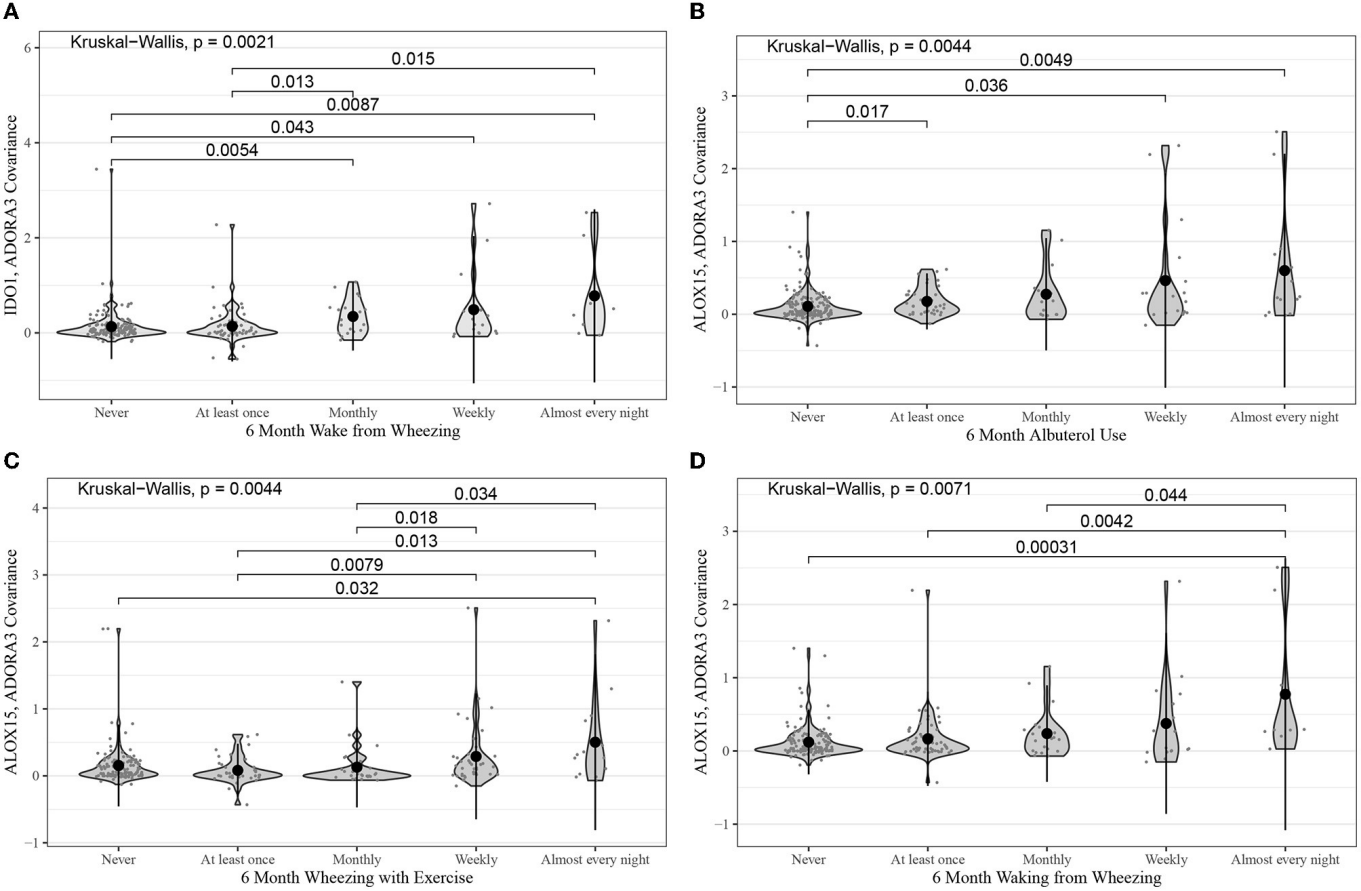
Violin plots for the most statistically significant gene-gene covariance measures (Equation 5) and 6-month ACT score components relationships for the ABRIDGE cohort, where each dot represents values for one patient. Kruskal–Wallis was used to test for global differences, and Wilcoxon signed-rank was used to test for pairwise differences. **(A)** *IDO1* and *ADORA3* covariance in 6-month frequency of waking from wheezing; **(B)** *ALOX15* and *ADORA3* covariance in 6-month Albuterol use; **(C)** *ALOX15* and *ADORA3* covariance in 6-month frequency of wheezing with exercising; **(D)** *ALOX15* and *ADORA3* covariance in 6-month frequency of waking from wheezing.

To further explore the relationship between co-expression of genes in modules A and B and asthma control, Kruskal–Wallis tests were performed to determine whether covariance measures for all possible pairs of these genes differ across levels of the ACT score components. Eight of the total 24 tests resulted in *p*-values less than 0.05, with the top six coming from module B. The most significant test involved the co-expression of *IDO1* and *ADORA3* and the frequency of waking from wheezing in the past 6 months (*p* = 0.0021; Figure 2).

### 3.2. ACDC in CAMP

We performed ACDC using data from CAMP in an attempt to replicate results observed for the top five modules identified in ABRIDGE. We found evidence of differential co-expression for module B (*p* = 0.0315) but not module A (*p* = 0.6823; Table 2). Also, evidence of differential co-expression was observed for gene pairs in modules C and D, which were not significant in ABRIDGE, Interleukin 5 Receptor Subunit Alpha (*IL5RA*) and Peripheral Myelin Protein 22 (*PMP22*) in module C, and Interleukin 17 Receptor B (*IL17RB*) and Interleukin 6 (*IL6*) in module D. Note that these results have not been adjusted for multiple testing.

**Figure 3:**
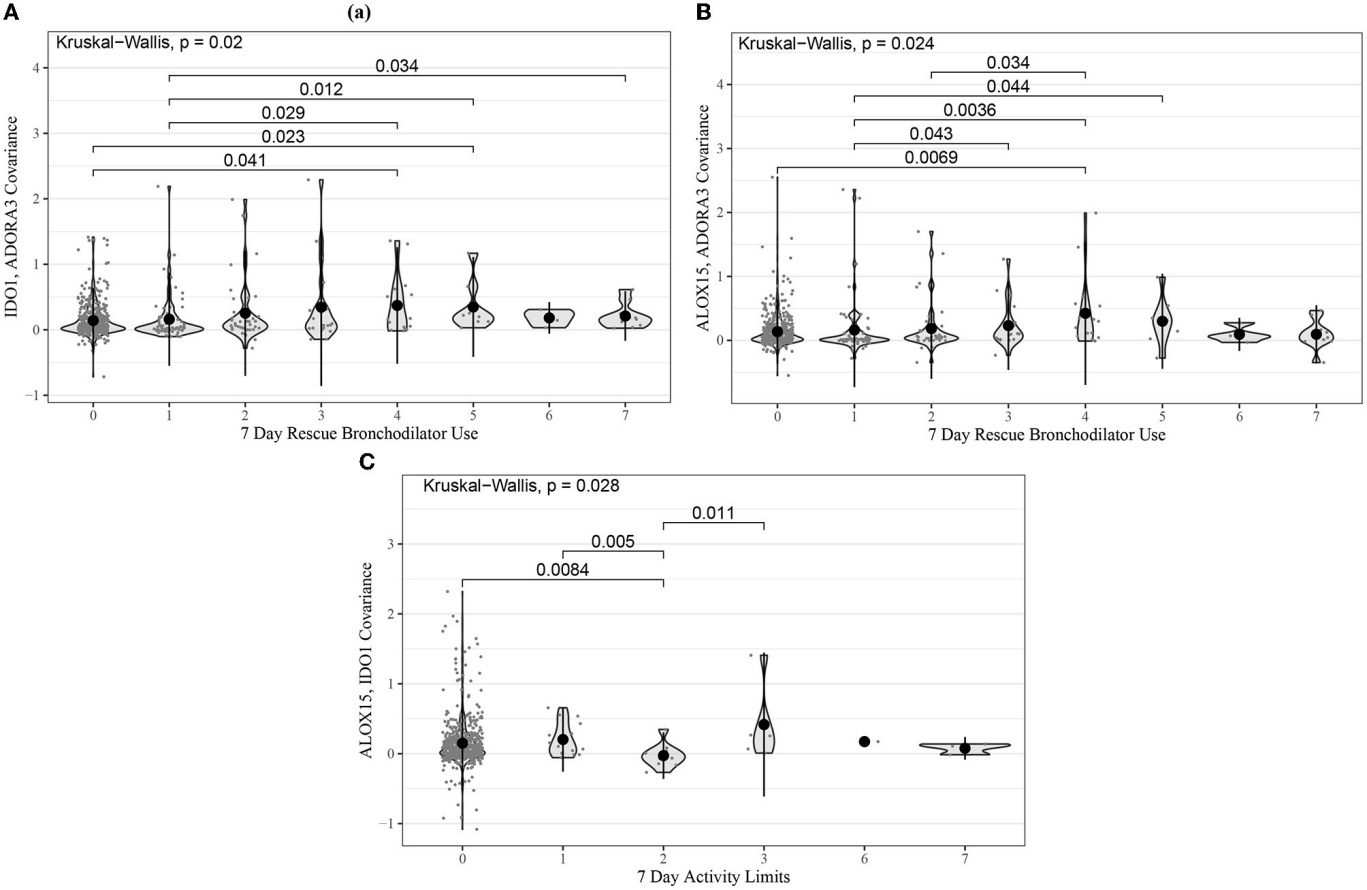
Violin plots for the most statistically significant gene-gene covariance measures (Equation 5) and 7-day ACT score components relationships for the CAMP cohort, where each dot represents values for one patient. Kruskal–Wallis was used to test for global differences, and Wilcoxon signed-rank was used to test for pairwise differences. **(A)** *IDO1* and *ADORA3* covariance in 7-day frequency of rescue bronchodilator use; **(B)** *ALOX15* and *ADORA3* covariance in 7-day frequency of rescue bronchodilator use; **(C)** *ALOX15* and *IDO1* covariance in 7-day activity limit.

Kruskal–Wallis tests were also performed for the same gene-pair covariances tested in ABRIDGE. Of the 24 tests performed, there were three with *p*-values less than 0.05, all from module B. The most significant test compared the co-expression of *IDO1* and *ADORA3* across levels of rescue bronchodilator use in the past 7 days (*p* = 0.02) (Figure 3). Additionally, we performed Kruskal–Wallis tests for all gene-pair covariances and 7-day ACT components for the three modules with CCA Wilks–Lambda *p*-values below 0.05. Of the 20 tests performed, the same three pairs from module B showed evidence of differential co-expression, but no others had *p*-values less than 0.05.

### 3.3. Differential expression in ABRIDGE

**Figure 4:**
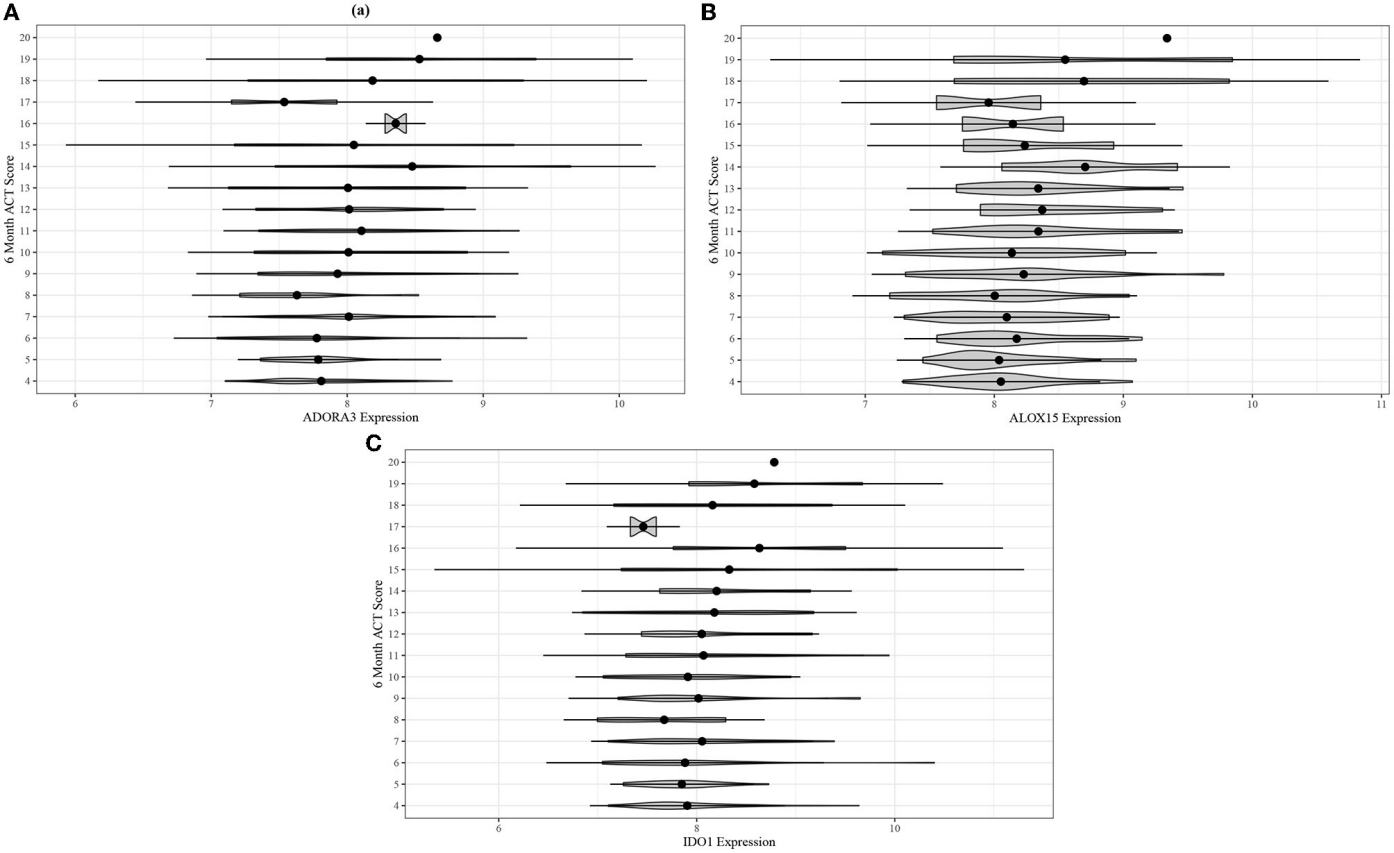
Violin plots for comparing unadjusted **(A)** *ADORA3*, **(B)** *ALOX15*, and **(C)** *IDO1* expression across 6-month ACT score levels in the ABRIDGE cohort.

Following the differential co-expression analysis, we performed ordinal logistic regression for each of the 13 genes in the top five modules and found increased risk of suboptimal acute asthma control for all genes in modules B and C, after adjusting for covariates (Table 3). Higher expression of *ADORA3, ALOX15*, and *IDO1* was associated with suboptimal 6-month ACT scores (Figure 4).

**Table 3:** Results of ordinal logistic regression models of genes in top five modules from CCA on ACT scores for ABRIDGE and CAMP cohorts.

		**ABRIDGE**			**CAMP**	
**Module**	**Gene**	**Odds ratio (95% CI)**	***p*** **-value**	**BH FDR** ***q*****-value**	**Odds ratio (95% CI)**	***p*** **-value**
A	*NLRP12*	1.3124 (0.7871, 2.1883)	0.2974	0.3634	1.2604 (0.7462, 2.1287)	0.3868
		*METRNL*	1.3057 (0.6326, 2.695)	0.4707	0.4707	1.2314 (0.6809, 2.2272)	0.4911
		*GHRL*	1.4324 (0.7231, 2.8375)	0.3028	0.3634	1.5659 (0.9256, 2.6491)	0.0945
B	*ALOX15*	2.3842 (1.4548, 3.9075)	0.0006	0.0017	2.4116 (1.7256, 3,3702)	2.54e^−7^
		*IDO1*	1.5986 (1.0665, 2.3962)	0.0231	0.0462	2.7940 (2.0725, 3.7667)	1.57e^−11^
		*ADORA3*	2.4457 (1.4882, 4.0192)	0.0004	0.0017	3.2442 (2.1922, 4.8010)	3.99e^−9^
C	*IL5RA*	2.6363 (1.3527, 5.138)	0.0044	0.0143	3.1781 (1.996, 5.060)	1.10e^−6^
		*PMP22*	2.2509 (1.4236, 3.5591)	0.0005	0.0027	3.8533 (2.5154, 5.903)	5.69e^−10^
D	*IL17RB*	0.5847 (0.1951, 1.7522)	0.3379	0.4816	0.9385 (0.353, 2.495)	0.8987
		*IL6*	0.9377 (0.253, 3.476)	0.9233	0.9233	0.5636 (0.1785, 1.780)	0.3284
E	*IL16*	0.7992 (0.451,1.4162)	0.4425	0.4816	1.0757 (0.6459, 1.7917)	0.7791
		*NLRC3*	0.5522 (0.212, 1.4382)	0.2240	0.4160	0.3926 (0.1864, 0.8268)	0.0139
		*SLAMF1*	0.5928 (0.2865, 1.2267)	0.1588	0.3440	0.9415 (0.4921, 1.8011)	0.8554

Models were adjusted for patient age, sex, race, data collection site, and the top three PCs.

### 3.4. Differential expression in CAMP

**Figure 5:**
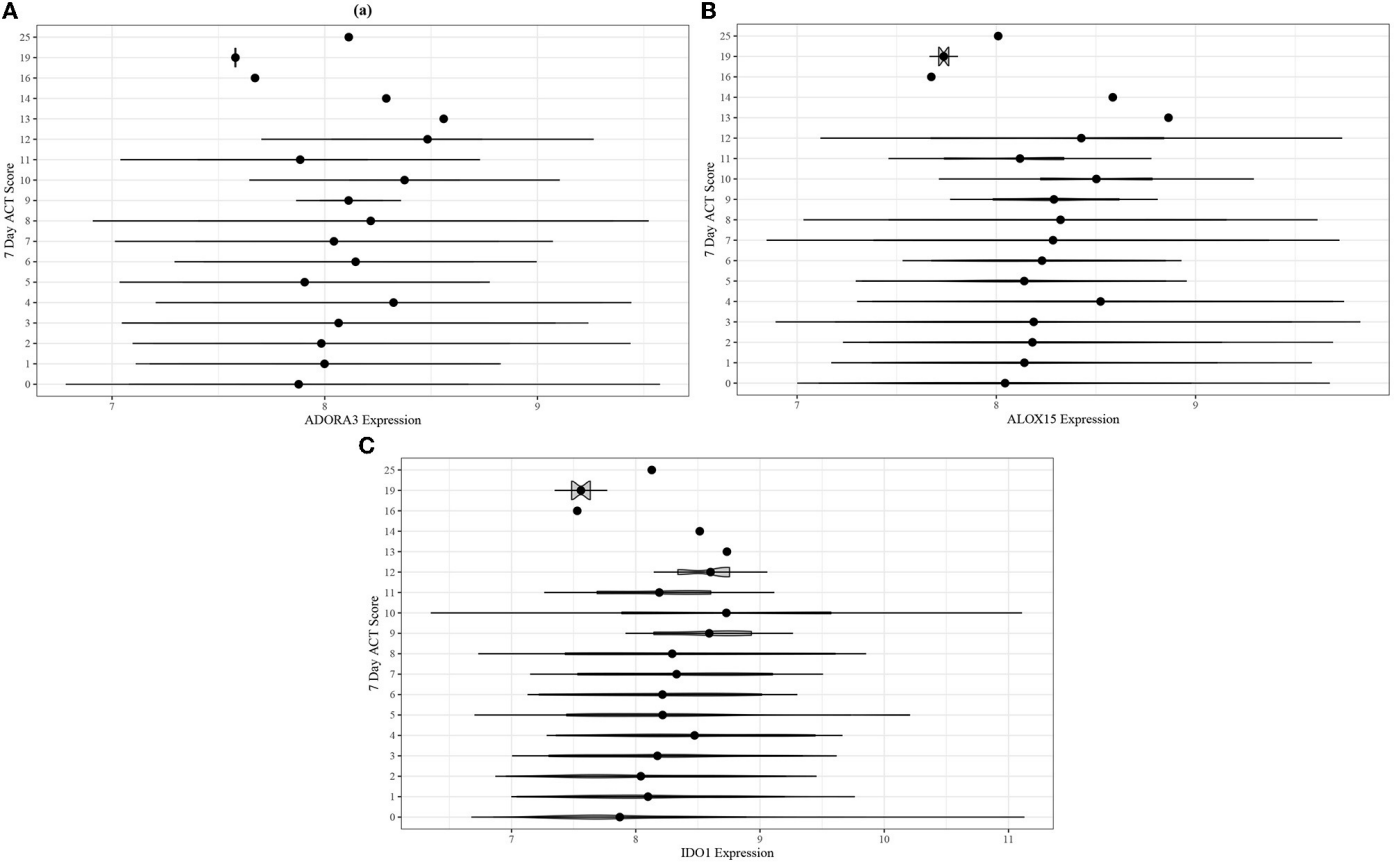
Violin plots for comparing unadjusted **(A)** *ADORA3*, **(B)** *ALOX15*, and **(C)** *IDO1* expression across 7-day ACT score levels in the CAMP cohort.

Adjusted ordinal logistic regressions were performed for the same 13 genes as the ABRIDGE cohort (Section 3.3). In the CAMP cohort, the regressions also showed highly statistically significant associations for all genes in modules B and C, and non-significant associations for modules A and D (Table 3). Unlike the results from ABRIDGE, a significant protective effect was seen for NOD-like Receptor Family CARD Domain Containing 3 (*NLRC3*) [OR: 0.3926, 95% CI: (0.1864, 0.8268)]. Associations between these genes and 7-day ACT scores (Figure 5) also imply that increasing gene expression is associated with suboptimal acute asthma control.

### 3.5. Methods comparison

The five most differentially co-expressed modules identified by the CoXpress analysis can be seen in Table 4. As a rule of thumb for identifying differentially co-expressed modules, the coXpress authors suggest pr_*g*_1__ ≤ 0.05 and pr_*g*_2__≥0.05, which implies correlations different than zero in one of the classes but not the other. None of the ABRIDGE modules met this threshold, and values of pr_*g*_1__, pr_*g*_2__ ≤ 0.05 for all of the five top modules indicate that the intra-module correlations are non-zero for patients with both better and worse asthma control. We note that genes *ADORA3* and *ALOX15* appear in module 1, the most differentially co-expressed module.

**Table 4:** Results of coXpress analysis in ABRIDGE whole blood gene expression dataset.

**Module**	**Genes**	***t*_1_**	***t*_2_**	**pr_*g*_1__**	**pr_*g*_2__**	** ${\bar{\text{corr}}}_{g_{1}}$ **	** ${\bar{\text{corr}}}_{g_{2}}$ **	**Mean difference**
1	*CCR3, ADORA3, ALOX15*	14.84	5.80	0.02	0.03	0.71	0.49	0.22
2	*CCL5, NKG7, ADA*	19.31	8.48	0.02	< 0.001	0.71	0.60	0.11
3	*TCIRG1, ADAM8, ZC3H12A, TNFAIP8L2*	31.82	11.07	< 0.001	< 0.001	0.68	0.58	0.10
4	*CTNNBIP1, ABCD1, EPHB6*	37.44	43.46	< 0.001	< 0.001	0.69	0.60	0.09
5	*SC11A1, IL1RN, IL1B, ALOX5AP, ALOX5, TLR6, FPR2, TLR8, MYD88, SIRPA*	70.59	38.03	< 0.001	< 0.001	0.72	0.63	0.08

*t*_1_ and *t*_2_ are the observed t-statistics in the better and worse control patient subgroups respectively, pr_*g*_1__ and pr_*g*_2__ are the probability of randomness statistics for the same groups, ${\bar{\text{corr}}}_{g_{1}}$ and ${\bar{\text{corr}}}_{g_{2}}$ are the mean pairwise correlation coefficients for the genes in the same groups and mean difference is the mean, pairwise difference between the correlation matrices for the groups.

## 4. Discussion

Here, we have described a novel approach to differential co-expression analysis that accommodates categorical, ordinal, or continuous exposures or outcomes. We suggest that co-expression features can be included in a linear modeling framework either as predictors or outcomes. To handle multivariate external features, we introduce ACDC, for either exploratory analyses or formal hypothesis testing. This strategy contrasts to most existing methods that test for differences in co-expression across a small number of classes. Another key difference is that identified modules can be small or large, which is not possible in many other methods. For example, DICER only accepts modules with at least fifteen genes (13). Although Partition was applied here to identify modules of correlated genes, other methods could be used, such as weighted gene co-expression network analysis (WGCNA) (14). Additionally, this framework can be applied to other types of molecular data, such as proteomics or metabolomics.

Application of the ACDC differential co-expression approach and ordinal logistic regression analyses identified three genes, *ADORA3, ALOX15*, and *IDO1* whose covariances and expression levels were associated with 6-month and 7-day ACT scores in the ABRIDGE and CAMP cohorts, respectively.

Adenosine is a nucleoside which exhibits increased production during periods of lung inflammation. Mediation is controlled through adenosine receptors like *ADORA3*. Previously, studies have shown that while single nucleotide polymorphisms (SNPs) of *ADORA3* loci are not associated with asthma (15, 16), *ADORA3* expression is associated with immunoglobulin E levels in whole blood samples of asthmatic patients (17) and is differentially expressed when comparing patients with severe asthma to controls (18).

*ALOX15* has both anti-inflammatory and inflammatory effects depending on its regulation and has been previously implicated in the development of inflammatory diseases, including asthma. A few studies have shown that *ALOX15* can be found in airway mucosa of asthmatic patients (19, 20), and another study found evidence of differential expression of *ALOX15* between controls and asthmatics (21). Additionally, one study found that haplotypic genetic variation at the locus for *ALOX15* is associated with asthma (22).

The best understood function of *IDO1* is it's role as an immunoregulator in cancer, inhibiting the body's ability to fight diseased cells, but its role in autoimmune responses is less clear. A mouse study showed that the entire indoleamine family promotes allergic airway inflammation (23), and a human study found evidence of differential expression of *IDO1* between patients with severe eosinophilic asthma, a more severe subtype of asthma typically found in adults and categorized by high peripheral blood concentration of eosinophils, and healthy controls (24).

Though all three genes have been previously identified as differentially expressed in asthma, there are varying degrees of understanding as to the biological roles that they play. To our knowledge, there are no studies that identify any of these genes as differentially co-expressed in asthma. This additional information could help to fill knowledge gaps about how the genes regulate or co-regulate asthma control.

A limitation of this analysis is the difficulty differentiating cause and effect between gene expression, acute asthma control, and medication use. Does gene expression affect response to asthma exacerbations or is it determined primarily by asthma control medications? The directionality of the relationship is particularly muddled by the inclusion of medication use in the calculation of ACT scores, which is standard practice (25).

The number of covariance features grows much more quickly than the number of genes in a module (or other gene set). Thus, for large modules it may be useful to reduce the dimensionality of the co-expression features or apply a feature selection mechanism in a preliminary step. We are working to implement two dimension reduction approaches: first, sparse CCA using elastic net penalized regression and second, applying Partition to the co-expression matrix. Additionally, the ability to adjust for covariates in the CCA step would add to the utility of the approach.

In the comparison analysis using coXpress, an existing and highly-cited module-based differential co-expression method, genes *ADORA3* and *ALOX15* were identified among the most important, but no modules reached statistical significance. To achieve statistical significance, coXpress requires that correlations be undetectable in one condition and detectable in the other. Kruskal–Wallis tests of the co-expression matrices showed differences in co-expression across levels of ACT, indicating that while co-expression is present at all levels of ACT, it is nevertheless different across levels. This type of relationship cannot be captured by coXpress. Also, to use coXpress, the ACT score must be dichotomized, which results in information loss.

Further study is needed to understand the larger network that includes *ADORA3, ALOX15*, and *IDO1*. All three are part of the Nakajima Eosinophil pathway, a group of the top 30 eosinophil-specific genes (26). This pathway is not well-studied and while much has been published about the role of eosinophils in asthma, few studies have looked at the role this pathway plays in asthma exacerbations or symptomology. More study is needed to determine what drives the associations with covariances observed here. They could be related to differences in the expression of eosinophil genes between eosinophilic and non-eosinophilic asthmatics. Alternatively, within eosinophilic asthmatics, within non-eosinophilic asthmatics or for all subtypes, covariances may be associated with symptom control. That is, differences in expression of eosinophil genes within some of these groups may be associated with symptom control or the associations may be driven by differences between groups.

In summary, we propose a novel strategy for differential co-expression analysis that is a flexible extension to prior methodology. In applications to ABRIDGE and CAMP cohorts, we find evidence of both differential co-expression and differential expression across ACT scores for *ADORA3, ALOX15*, and *IDO1*, all genes which have been previously implicated in asthma. These genes may be involved in the underlying regulatory mechanisms behind acute asthma control, however, further study is needed.

## Data availability statement

The CAMP dataset can be found in the Gene Expression Omnibus (GEO; https://www.ncbi.nlm.nih.gov/geo/) repository under accession number GSE22324. The ABRIDGE data is being submitted to GEO and will be available as soon as that process is complete.

## Ethics statement

The studies involving human participants were reviewed and approved by Partners Human Research Committee. The patients/participants provided their written informed consent to participate in this study.

## Author contributions

The differential co-expression approach was conceived by KQ and JM. The analysis was conducted by KQ. All authors contributed to interpretation of the results and writing of the manuscript. All authors contributed to the article and approved the submitted version.
