# Supplementary material for: ACDC: a general approach for detecting phenotype or exposure associated co-expression
Source: Front Med (Lausanne). 2023 May 19;10:1118824. doi: 10.3389/fmed.2023.1118824 (PMC10235619; doi:10.3389/fmed.2023.1118824)
Supplement: Supplementary file 1 [file Data_Sheet_1.pdf]

Supplementary Material

1 SUPPLEMENTAL INFORMATION

1.1 OSCA

OmicS-data-based Complex trait Analysis (OSCA) is a suite of C++ functions that provides an estimate of the percent of variance in an external phenotype that can be explained by an omics profile, akin to heritability estimates in GWAS. Here, we use OSCA's Omics Restricted Maximum Likelihood (OREML) method to determine the percent variance explained in 6-month ACT scores by increasingly reduced gene expression data. These results can be seen in Figure S2 and Table 4.

For the ACDC analysis, we aimed to choose a dataset that balances within and between-module variance and reduces noise and thus, chose an ILC of 0.35, corresponding to 53% reduction in features. This dataset explains 12.2% of the variance in 6-month ACT score by between-module relationships. Therefore, the 10% percent variance explained that seems to be loss by reducing the data 53% is contained within the modules created at this level of reduction. Additionally, we see that the 72% reduced dataset explains roughly the same variance in ACT score, however by the nature of Partition, these modules are larger and thus, noisier.

2 SUPPLEMENTARY FIGURES

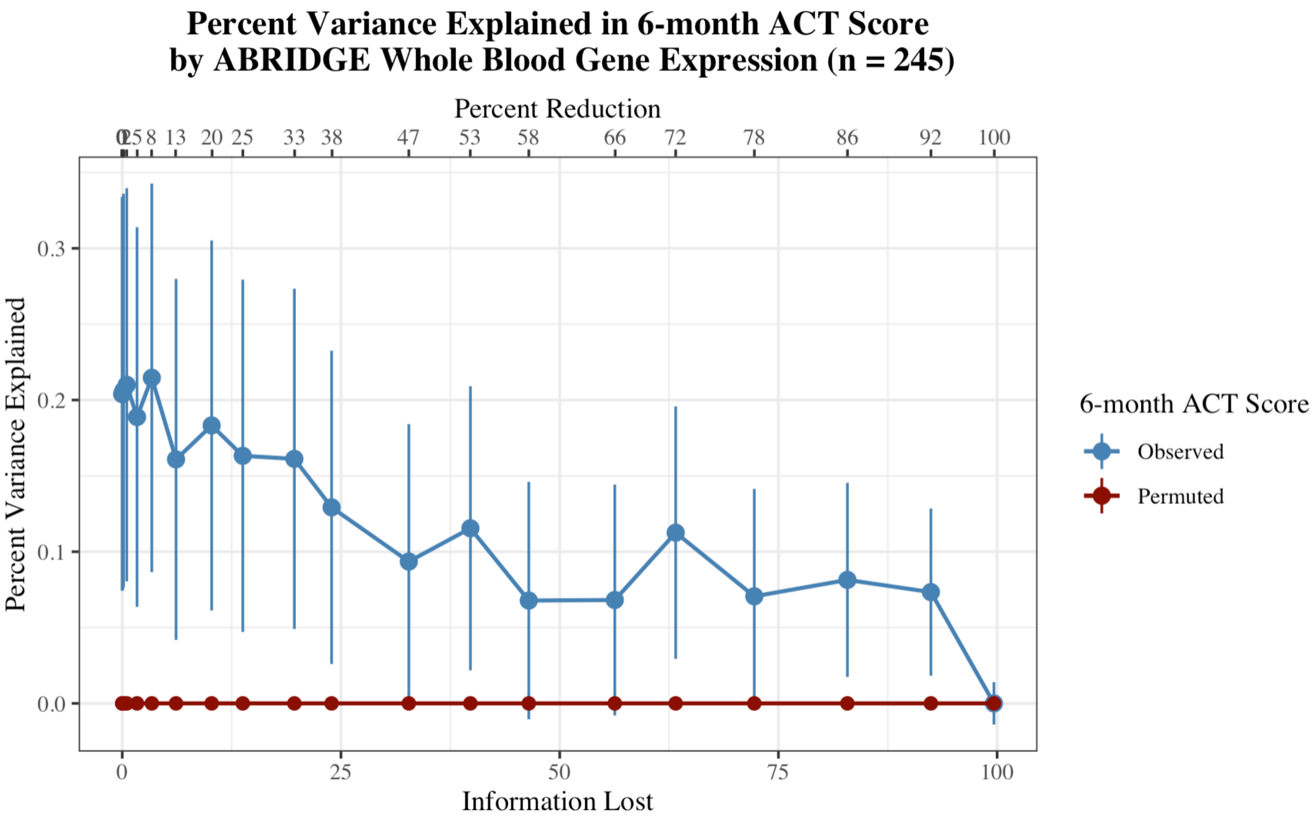

Figure S1. Plot showing percent variance explained in 6-month ACT score by ABRIDGE whole blood gene expression data at varying level of dataset reduction, calculated for observed phenotypes in blue and permuted phenotypes in red. An information loss value of 0 represents the unreduced dataset, and an information loss level of 100 represents the data being reduced to the average expression of all genes.

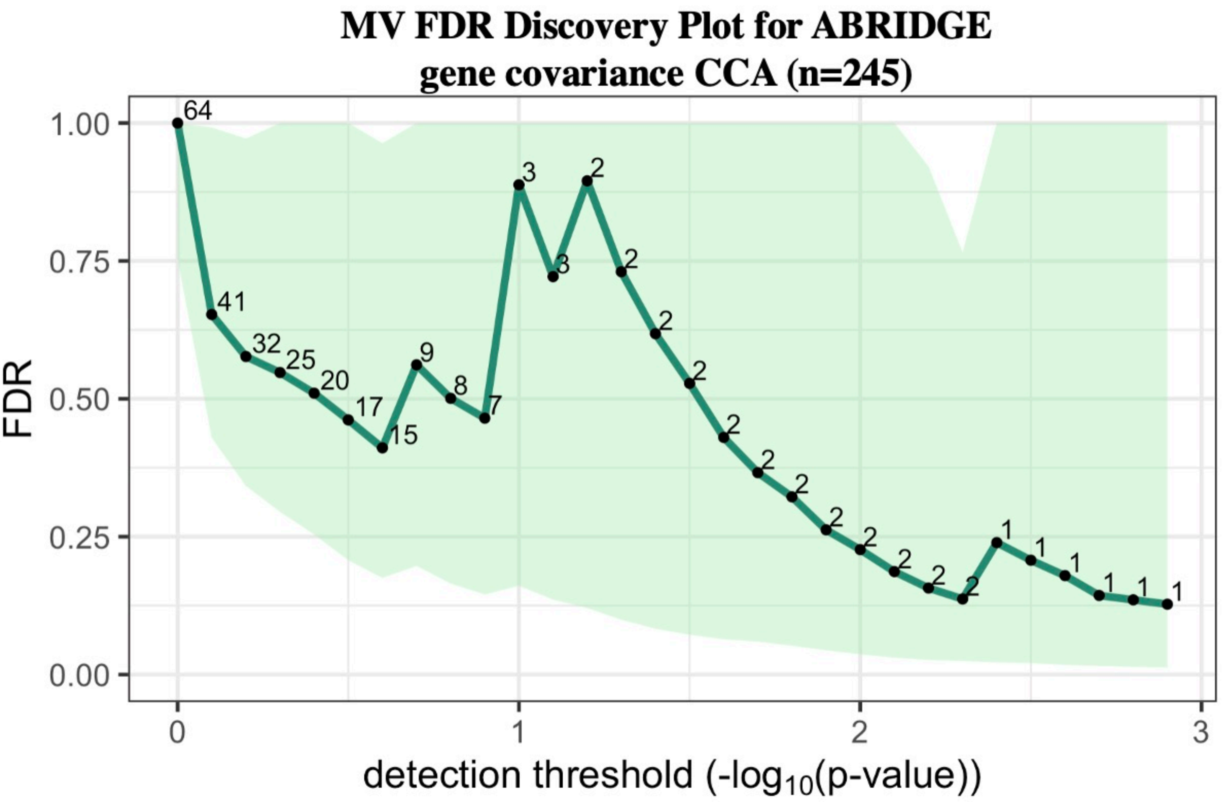

Figure S2. MV FDR discovery plot for CCA between ABRIDGE gene-gene covariance measures and ACT score components. After 250 permutations, likelihood ratio test p-values were computed for both the permuted and non-permuted data across a series of possible detection threshold. The numbers above the FDR estimate line denote the number of discoveries at each threshold, and the green shaded area is the 95% CI for the FDR estimate.
